# Good quantification practices of flavours and fragrances by mass spectrometry

**DOI:** 10.1098/rsta.2015.0365

**Published:** 2016-10-28

**Authors:** Frédéric Begnaud, Alain Chaintreau

**Affiliations:** Firmenich SA, Corporate R&D Division, Route des Jeunes 1, CH-1211 Geneva 8, Switzerland

**Keywords:** mass spectrometry, good practices, multi-analyte, identification points, quantification, validation

## Abstract

Over the past 15 years, chromatographic techniques with mass spectrometric detection have been increasingly used to monitor the rapidly expanded list of regulated flavour and fragrance ingredients. This trend entails a need for good quantification practices suitable for complex media, especially for multi-analytes. In this article, we present experimental precautions needed to perform the analyses and ways to process the data according to the most recent approaches. This notably includes the identification of analytes during their quantification and method validation, when applied to real matrices, based on accuracy profiles. A brief survey of application studies based on such practices is given.

This article is part of the themed issue ‘Quantitative mass spectrometry’.

## 1. Introduction

Gas chromatography-mass spectrometry (GC-MS) has been the gold standard for the identification of natural ingredients since the infancy of the technique in the 1960s [1]. Until the 2000s, the quantification needs of the flavour and fragrance (F&F) domain were rather modest, with few constraints on final accuracy. Only classic quantification techniques were required, such as GC hyphenated to flame ionization detection (FID) and sometimes to MS, with a focus on precision rather than accuracy. Liquid chromatography-MS (LC-MS) was not a typical quantification tool. The only well-developed quantitative field in F&F dealt with the naturalness of flavour ingredients by isotopic MS, which does not fall within the scope of the present article [2].

New constraints occurred, however, with emerging regulations, mainly in Europe. The first event arose in 1999, with the publication of opinion by the Scientific Committee on Cosmetic Products and Non-Food Products (SCCNFP) on fragrance allergens [3]. It led to a European regulation in 2003 that required the labelling of 24 volatile fragrance compounds (electronic supplementary material, table SM-1) when they occurred at above 10 mg kg^−1^ in ‘leave-on’ consumer products, i.e. remaining on the skin [4]. As a consequence, these compounds had to be quantified down to this concentration with a known accuracy in formulae containing tens of other volatile ingredients, frequently representing much more than a hundred GC peaks. Two years later, the Scientific Committee on Consumer Products (SCCP) published an opinion on the potential phototoxicity of 15 furocoumarins (electronic supplementary material, table SM-2) occurring in several essential oils and plant extracts [5]. In 2008, a European regulation implemented a restriction of 11 biologically active substances in food leading to the GC-MS monitoring of eight of them in flavours (electronic supplementary material, table SM-3) [6]. The adoption of REACH (Registration, Evaluation, Authorization and Restriction of Chemicals) by the European Parliament in 2006 [7] created a number of quantification needs to support the biodegradability and ecotoxicology tests of fragrance ingredients. The last major event occurred with the recent opinion of the Scientific Committee on Consumer Safety (SCCS), formerly SCCNFP, which proposed increasing the number of chemically defined fragrance allergens to be monitored from 24 to 54 (electronic supplementary material, table SM-1) [8]. In general, a new paradigm has emerged in F&F analysis over the last 15 years: the quantification methods developed to meet the new regulations demand proven results in the case of debate between concerned parties, including the authorities. As a consequence, not only do these methods need to be built on good analytical practices, but they must also be validated according to the highest standards.

All these new rules created an analytical challenge for the different partners of the F&F chain: the raw material suppliers, the fragrance and cosmetic industries, and the official or contract laboratories. In addition, although the latter could analyse hydrophilic and non-volatile pharmaceutical compounds, they had little or no experience with volatile and hydrophobic fragrance ingredients, for which no method existed. The development of multi-analyte quantification techniques became compulsory in order to monitor so many analytes in a reasonable time frame. This raised new challenges in terms of selectivity and specificity of instruments requiring chromatographic separation of analytes hyphenated to a selective detection method, such as MS. One major objective was to avoid interferences between one given analyte and the others, and, as much as possible, interferences between the analytes and the matrix constituents. The second major objective consisted of distinguishing the analyte being measured from other co-eluting or overlapping compounds of the matrix, which is a frequent situation in perfumes and flavours, as they are often composed of more than a hundred constituents. In addition, the fact that such quantifications had to meet regulations implied that their reliability in complex F&F media had to be numerically evaluated. Therefore, the guidelines and norms related to the validation of analytical techniques had to be applied not only to assess this reliability, but also to prevent the use of a multitude of methods from studies that involved poor instrumental set-up or quantification practices (e.g. [9–13]).

## 2. Basic principles of flavour and fragrance quantification

### (a) Preliminary precautions

The following recommendations are crucial to ensure reliable quantification, but they do not fall exclusively within MS methodology, and so we invite the reader to refer to the articles cited below for detailed procedures. Although this article focuses on technical practices, one must keep in mind that quantification has to be conducted by trained analysts who understand the rationale behind the present recommendations.

#### (i) Suitability of the instrumentation

The instrument used for quantification should be tested prior to performing the quantification in order to limit and stabilize the associated experimental error. Suitability tests according to the manufacturer's specifications are advisable, but this does not preclude the use of internally defined standards adapted to the F&F domain, particularly when dealing with labile or sensitive compounds. The chromatographic system should be tested for efficiency, resolution and adsorptions, and the MS system should be tested for source adsorption and acidity (the absence of dehydration), mass accuracy and abundances [14,15]. The MS detector response should preferably be linear [16] or exhibit a low curvature that can be checked by using one-way analysis of variance. In the latter case, the analyst has to check that the measured concentration is proportional to the analyte concentration with a null offset and a slope equal to unity (method linearity). The response curve must never be forced to zero because of a possible residual signal due to the matrix background. The so-called zero value, i.e. the response of a blank matrix, should always be measured. Its relevance can be statistically checked by using a *t*-test.

#### (ii) Purity assessment of internal and calibration standards

The easiest way to obtain pure standards is to purchase them with certified identity and purity. However, chemicals can deteriorate over time, or not be commercially available as reference materials, and then their purity must be (re-)assessed. The gold standard lies in the use of ^1^H-NMR with a certified internal standard (IS). It is applicable both to volatile and non-volatile compounds with an accuracy of about 1%, and it simultaneously allows confirmation of the identity of the compound [17,18].

NMR is not available in all laboratories, however. As a more handy, but less accurate, alternative for volatile compounds, GC-FID analysis can be performed by using a certified IS and predicted response factors [19]. It allows estimation of purity with a mean accuracy of 6%. It must be recalled that, when no IS and no response factor is used, the non-volatile compounds in a mixture of volatiles are overlooked. Therefore, raw FID percentages cannot be applied to purity measurements [20]. Instead, this undetected amount is evaluated by using the predicted response factors.

#### (iii) Sample preparation

Sample preparation necessarily induces the addition of experimental errors that must be minimized as much as possible. The suitability of all instrumentation used to prepare a sample has to be established (balance, volumetric flasks, volume dispensers, etc.). In some cases, the direct analysis of F&F samples is achievable without any sample treatment, except dilution or filtration (e.g. alcoholic perfumery, compounded fragrances and flavours if the amount of non-volatile constituents is low when submitted to GC). However, if the analytes occur in more complex media, such as emulsions, cosmetics and foods, they need to be extracted from their matrix. Isolating the volatile fraction for GC-MS can notably be achieved by solid-phase microextraction [21,22], simultaneous distillation–extraction [23] or headspace extraction [24]. For complex samples, one of the most popular techniques is solid-phase extraction [25], which is often used prior to LC-MS measurement [26–28].

We emphasize the fact that several sample preparation techniques lead to non-quantitative recoveries [29]. It is therefore important to evaluate these recoveries and to validate this step, either independently or together with the final validation of the quantification method.

#### (iv) Blanks

Because carry-over issues are frequent in trace analysis, particularly when using an LC injector, the recommendation is to optimize the rinsing steps of the autosampler and to run blanks between all calibration and sample injections during the development stage. Afterward, the number of blanks can be reduced at the application stage, after the absence of carry-over has been observed. Different blanks should be considered on the basis of the analytical constraints (solvents used, matrices, presence of IS).

### (b) Analyte identity

‘Confirmation of identity should be objective and reliable, not depending on the subjective interpretation of the operator’ [30]. F&F matrices are generally complex, leading to a significant risk of co-elution between the peak of interest and interfering compounds exhibiting a spectrum with similar ions (for sesquiterpenes, for instance). To minimize this risk, it is crucial to enhance the selectivity of the separation method and the specificity of the detection means, which will favour unambiguous identification of chromatographic peaks with the MS quantification signal. The European Commission has adopted a decision on the performance of analytical methods that solves this problem [31]. It consists of the use of ‘identification points’ (IPs). MS techniques used in quantification often generate spectra with few fragments (selected-ion monitoring (SIM), chemical ionization MS, LC-MS, etc.), except for specific techniques that are currently marginally used but that may expand in the near future (orbitrap, time of flight). In the case of co-elution, when the acquisition is made in full scan, only a few fragments may come exclusively from the target analyte and can be used. Therefore, the peak identification cannot be performed with the usual algorithms applied to the recognition of full spectra. Deconvolution algorithms are useful for identification purposes, but their quantitative reliability has never been formally evaluated. Consequently, their result cannot provide the analyst with an IP. The IPs derive from the use of ratios between the abundance of target ions, and they should fall between tolerance intervals defined in the European directive (table 1). Examples of IPs earned by various MS techniques are given in the electronic supplementary material, table SM-4. Such identification criteria are already implemented in pharmaceutical, veterinary and forensic analyses [30,32–34] and were more recently adopted by the International Organization of the Flavor Industry (IOFI) for the identification of flavouring substances in nature [35]. As a general rule, the positive identification of the target analyte requires that four IPs be obtained, as detailed hereafter for GC- and LC-MS, if the chromatographic resolution is not taken into account (table 2).

**Table 1.** Tolerances for abundance ratios [31]. (EIMS: electron-impact MS; CIMS: chemical ionization MS).

relative intensity (% of base peak)	accepted deviation in GC-EIMS (%)	accepted deviation in GC-CIMS, GC-MS*^n^*, LC-MS*^n^* (%)
>50	10	20
20–50	15	25
10–20	20	30
<10	50	50

**Table 2.** Examples of IPs per ion (for full details, see [31]).

MS technique	IPs per ion
low-resolution MS	1
precursor ion from QQQ	1
product ion from QQQ	1.5
high-resolution MS	2

However, applying the IPs manually as described above is time-consuming and its automation is not implemented in the workstations of all MS suppliers. To speed up data treatment, or to automate it (see the next section), characterizing the peak identity with a single numerical descriptor may be useful. Agilent Instruments has long proposed checking for the peak identity by calculating its associated *Q* value (electronic supplementary material, equation SM-1). It can easily be programmed and gives identification results similar to those with the use of IPs (A.C., unpublished results).

### (c) Specific gas chromatography-mass spectrometry features

The use of an IS is compulsory for a syringe injection because of the low repeatability of injected volumes. For headspace or solid-phase microextraction injections, internal standardization is often unsuitable and external standardization is generally recommended, except if a labelled IS is used. In all cases, an isotopomer of the analyte is the best choice as an IS. General guidelines of GC quantification methods can be found elsewhere [36], and the quantification in SIM is described in an IOFI guideline [37].

The European directive indicates that the chromatographic retention time (RT) of an analyte, relative to that of its IS, should fall within less than 5% of the relative RT of the reference compound [31]. In the specific case of capillary columns with bonded phases, we have observed much better repeatability of these relative RTs, even in complex matrices (A.C., unpublished results). Therefore, we consider that, if the relative RT bias is less than 2%, this is equivalent to one IP, and three additional IPs are required when using MS to confirm the identification. If a quadrupole MS (Q or QQQ) is used, it can be operated (i) in full-scan mode from which the specific ions of the analyte are extracted, (ii) in SIM mode, (iii) in chemical ionization mode, or (iv) in tandem (MS/MS) mode. In general, partial spectra with only a few fragments are obtained, to which the IP calculation is applied. It is also advisable to apply the IP calculation to the ions extracted from full spectra. The auto-ionization and adduct formation trend of old hyperbolic ion traps has been observed, and such a risk should be carefully investigated by using a suitable robustness test because biased results have been reported, notably in the context of a ring test [38–40].

### (d) Specific liquid chromatography-mass spectrometry features

In high-performance LC (HPLC), the usual column lengths correspond to very low peak capacities compared with GC, and so the RT is never a sufficient identification criterion. Even columns packed with a sub-2 µm diameter stationary phase combined with optimized ultra-high-pressure LC do not compete with the resolution of GC capillary columns, except in unusual cases not yet applied to the F&F flavour domain [41]. Therefore, the LC-MS identification has to be supported by four IPs. As a consequence, a single quadrupole can never be suitable for multi-analyte quantification.

## 3. Data processing strategy and validation

### (a) Decisional tree

Combining the raw quantitative data with the identification results may lead to complex rules for a routine laboratory. The interpretation of results may be made easier for the analyst's task with the help of a decisional tree (electronic supplementary material, figure SM-1). Such a decisional tree becomes compulsory to clarify the logic of the data treatment when it must be translated into an automation program.

### (b) Automation

**Figure 1.**
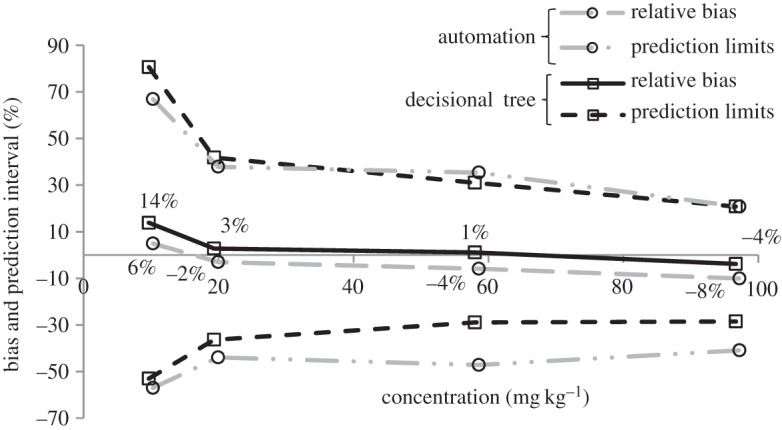
Prediction interval at 90% confidence, using the application of the decisional tree and the corresponding automation (GC-MS quantification of 24 allergens) (reprinted from Chaintreau *et al*. [42], with permission of Elsevier).

The interpretation of all results generated by multi-analyte quantification is time-consuming, and the automation of this step can become essential to ensure correct throughput of the laboratory. In the example of figure 1, this interpretation has been computerized, starting from the corresponding decisional tree. This automation should itself be validated and compared with the data treatment, such as in this example, to assess whether it performs similarly to the analyst's interpretation.

### (c) Validation

As proposed by guideline ISO17025: ‘Validation is the confirmation by examination and the provision of objective evidence that the particular requirements for a specific intended use are fulfilled’ [43]. Validation must include a clear specification of the requirements, and it must give the experimenter and the receiving party guarantees that every single measure that is routinely performed will be similar to the unknown true value of the sample, within a measured and proven accuracy range. Most of the work has been triggered by the pharmaceutical industry, and since the first publication of rules and guidance, numerous standards have been published by normative associations [16,43–45]. They define the vocabulary and the statistical tools required to validate a method. The main validation criteria commonly used in analytical laboratories include selectivity, response function (calibration curve), method linearity (nominal concentration versus measured concentration), accuracy (=trueness and precision, i.e. repeatability and intermediate precision), limit of detection (LOD), limit of quantification (LOQ), assay range, sensitivity and robustness. Other specific criteria can be required such as analyte stability and recoveries. All these criteria are matrix dependent, and ideally, they must be evaluated in the matrix, or at least in a medium that mimics the matrix. To this extent, the expertise of analysts is essential because they are responsible for evaluating the similarity between matrices that will be met in future samples.

The validating method also eases the transfer within a laboratory network by establishing clear, measureable and comparable endpoints between laboratories. One must keep in mind that if a balance has to be found between costs, technical feasibility and associated risk, no compromise must be made on the technical/chemical side. Validating a method is not proof of its reliability from a chemical viewpoint, and an interfering reaction (hydrolysis, oxidation and photodegradation) may impair the result by affecting the robustness of the method.

#### (i) Confidence interval and tolerance interval

The confidence interval is a conventional statistical calculation allowing determination of the interval into which the true value of a measured parameter will fall. More practically, for replicate measurements, it corresponds to the interval where the average of a series of determinations will fall, if done by the same number of participants:
3.1
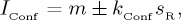

where *m*: mean measured value at a given concentration; *k*_Conf_: coverage factor for the confidence interval; *s*_R_: standard deviation of the intermediate precision.

It is extensively used but is of limited interest for the analyst in day-to-day work, where generally no replicate measurement is conducted. To determine the acceptance range in which one new measurement will fall, then the prediction interval, *I*_Pred_, has to be determined. This interval is by essence equal to or larger than the confidence interval. It requires a slightly different calculation, essentially concerning the coverage factor *k*_Pred_ (see details in electronic supplementary materials):
3.2
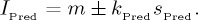


This approach is used to establish the accuracy profile, as depicted in figure 1.

#### (ii) Accuracy profile

**Figure 2.**
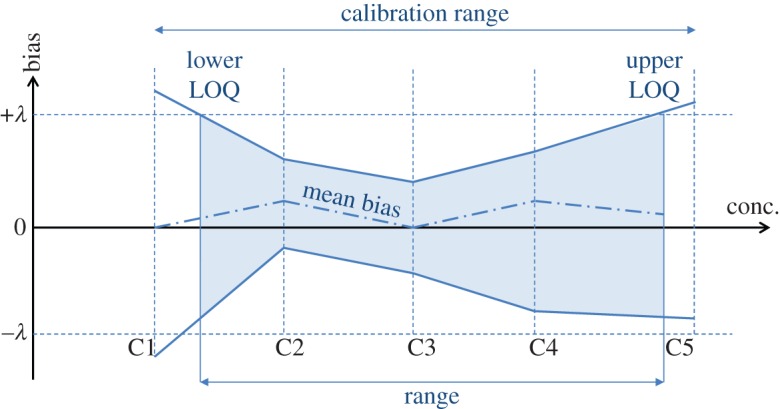
Scheme of an accuracy profile. C1–C5 = calibration concentrations; *λ *= tolerance interval, previously set by the requester; LOQ = limit of quantification (adapted from Feinberg *et al*. [47]). (Online version in colour.)

Among the different approaches, the accuracy profile combines both a rigorous statistical data processing [43,46] and output that is directly applicable to day-to-day use in an analytical laboratory (figure 2). It particularly avoids the frequent issue of acknowledging an accurate average, despite poor precision (=mean value close to the target, with a high dispersion of individual measurements) [48,49].

By considering the combination of both trueness and precision (=total error), the accuracy profile defines an interval *I*_Pred_ (equation (3.2)) in which a known proportion of measurements will be found (details in electronic supplementary material). This approach has been extensively published [50,51] and commercial software to automate the experimental design, data processing and reporting are available in good laboratory practices environment [52].

**Figure 3.**
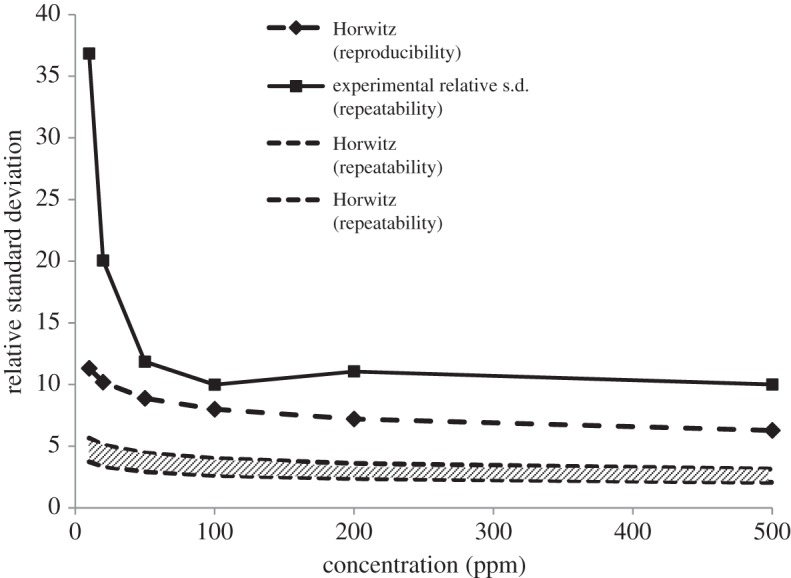
GC-MS quantification of 24 allergens: comparison of experimental repeatability (relative s.d.) with the repeatability and reproducibility predicted by the Horwitz equation (adapted from Bassereau *et al.* [57]).

Validation samples have to be established by using a matrix representative of the sample matrix. Consequently, the matrix effect is intrinsically taken into account when measuring all necessary endpoints, notably the LOD and LOQ, which is of critical importance for complex matrices. This avoids the publication of appealing but inapplicable results when using the unrealistic determination of these important characteristics: by visual estimation, as a multiple of the signal/noise ratio, from the standard deviation of a blank or from the regression parameters of the calibration line at low concentration [53–55]. These techniques only give estimates of the LOQ and are generally over-optimistic and inapplicable for routine analysis. By contrast, when based on accuracy profiles, the LOQs correspond to the lowest and highest concentrations where the tolerance line crosses the accuracy profile limits (figure 2). In the same vein is the recommendation of the European directive that ‘the inter-laboratory coefficient of variation (CV) for the repeated analysis of a … fortified material, … shall not exceed the level calculated by the Horwitz Equation’ [31]. However, this equation results from a compilation of existing experimental results reporting inorganic analyses and gathered from the literature between 1975 and 1985 [56]. Therefore, it is not representative of the variability in results in the case of complex organic mixtures, as illustrated in figure 3, and so it may be acceptable for the experimental CV to be higher than Horwitz's prediction.

**Figure 4.**
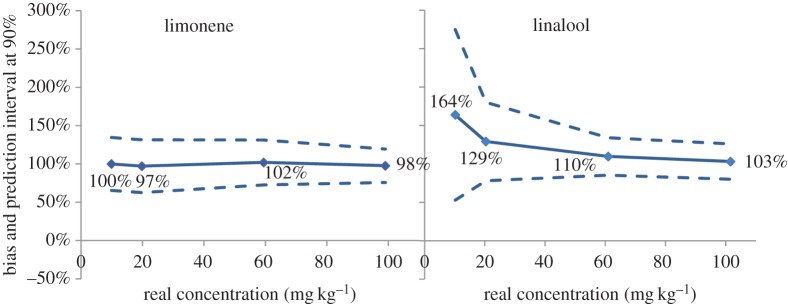
Prediction interval (*α* = 90%) resulting from the validation of GC-Q quantification of 24 allergens by 10 laboratories (adapted from Chaintreau *et al.* [42]). (Online version in colour.)

The most significant advantage of the accuracy profile is the evaluation of the performances of a method over the entire validation range. LOQ can be adapted on the basis of the tolerance interval defined by the analyses requester, and interpolation allows identification of LOQ that may not necessarily correspond to a validation point. This is particularly useful when the analytes are submitted to a limit of declaration or a limit of use by the regulation. For instance, if a fragrance allergen occurs in a consumer product that is not rinsed from the skin, its occurrence must be declared if higher than 10 ppm. In the example of figure 4, considering the prediction interval we observe that it corresponds to a reasonable range at low concentration for limonene, in contrast with that of linalool. However, the large interval of the latter must be accepted and kept in mind because it meets a legal limit. More generally, a valid concentration domain (lower and upper limits) can be identified with this approach.

### (d) Robustness

According to ICH [44], ‘the robustness of an analytical procedure is a measure of its capacity to remain unaffected by small, but deliberate variations in method parameters and provides an indication of its reliability during normal usage’. The measurement of the robustness of the method relies primarily on the identification of potential sources of result deviations and the measurement and weighting of their effect by using an appropriate experimental design. Despite not yet being part of the validation in F&F applications, because of the huge variability of the possible matrices, ensuring a proper robustness study can be highly beneficial to ease the transfer between laboratories (the next step being the ruggedness, not detailed here). So far, however, we have not found any published GC-MS or LC-MS examples of robustness studies applied to the F&F domain.

#### (i) Ion suppression/enhancement

**Figure 5.**
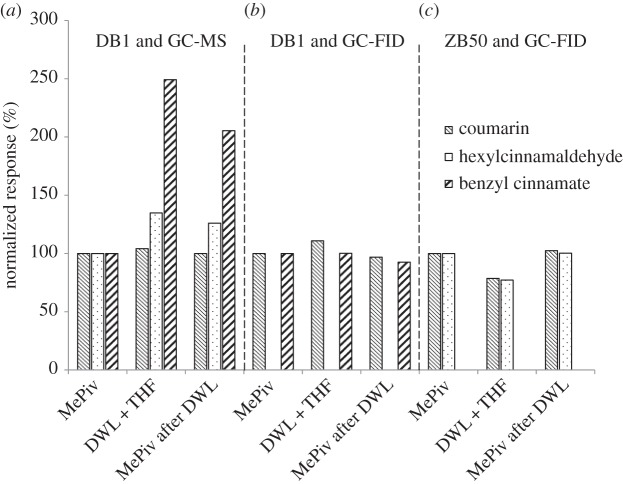
(*a*–*c*) Influence of matrix constituents on the normalized response after split injection (samples spiked with 80 mg l^−1^ of three suspected allergens; calibration in methyl pivalate and sample extracts in THF; Agilent instrument with MS or FID detection). DWL, dishwashing liquid; MePiv, methyl pivalate; THF, tetrahydrofuran (adapted from Chaintreau *et al.* [61]).

Although ion suppression is a well-known phenomenon [58], ion enhancement also occurs, but has been rarely documented in the literature [59]. Mastovska *et al*. previously proposed the use of polyhydroxylated substances to magnify the response of pesticides in GC-MS [60], as they thought that they interacted with the active sites of the GC column. More recently, we also observed important signal magnifications when injecting crude extracts of cosmetics and detergents via thermal desorption [61]. Because this occurred only with MS detection and not with FID (figure 5*b*), we assumed that the phenomenon took place in the MS source. Consequently, when such a peak magnification caused by the matrix constituents occurs, internal standardization is not applicable and a standard addition is required.

As a rule of thumb, calibrating into a blank real matrix is the most reliable strategy whenever possible [62].

## 4. Applications

Because the validation approach based on accuracy profiles was proposed in the 1990s [46] and the European guidelines on the performances of analytical method were issued in 2002 [31], the studies that applied the present recommendations were published only after 2002. In fact, because of their novelty, published applications combining these approaches in the F&F domain remain scarce.

### (a) Gas chromatography-mass spectrometry

#### (i) Regulated skin allergens

The present approach has been applied for the assessment of the European Norm [63], starting from the GC-MS method published by the International Fragrance Association (IFRA) [64] and validated by the Centre Européen de Normalisation [42]. The biases and LOQs were determined in real matrices after identification of analyte peaks by using *Q* values, and the method linearity was checked. Although this method only applied to ready-to-inject samples, a variant that includes online sample clean-up was developed for fragranced cosmetics and detergents with the same approach and validated as a whole [61]. From the headspace sampling of cosmetic extracts, another GC-MS method was also validated by using the accuracy profiles [65]. However, the analytes were consecutively identified and quantified from two independent injections, one in full scan and one in SIM mode, whereas both should be made in the same run. For the extended list of 54 allergens, a two-dimensional GC-MS approach has been proposed and validated by the determination of accuracy profiles [66]. All other published methods did not fully apply good quantification practices or a full validation by using spiked real samples.

#### (ii) Atranols, musks, bioactive flavour compounds, contaminants

None of the methods related to these compounds meet the present recommendations.

### (b) Liquid chromatography-mass spectrometry

#### (i) Regulated skin allergens, atranols, furocoumarins, contaminants and musks

**Figure 6.**
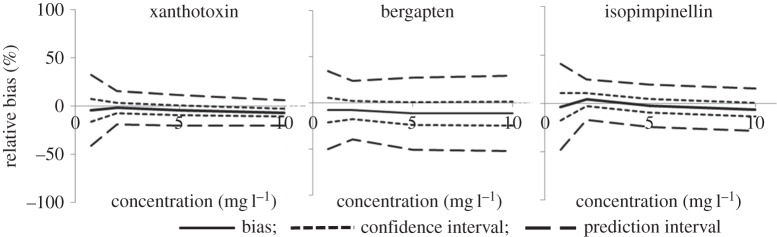
Mean bias, confidence interval and prediction interval of three furocoumarins by 10 laboratories with HPLC-MS/MS. Mean biases ≤ 12% (with permission of IFRA).

To our knowledge, no work has yet been published in the F&F domain in which the present guidelines have been fully applied. Some studies still propose simple LC-MS despite its insufficient selectivity. When LC-QQQ in multiple-reaction monitoring mode is used, the validation of analyte identities by their ion ratios is always unclear, and the full validation by accuracy profiles has never been applied. For the furocoumarins, the initial LC-MS/MS method published in 2004 [67] was reworked by IFRA by using LC-QQQ and was a fully validated approach (figure 6), but was not published. A variant based on HPLC-exact mass MS also meets the present recommendations [68].

## 5. Conclusion

The development of a quantification method should combine good MS quantification practices with appropriate validation. Both parts form a whole to ensure reliable results. Whatever the sophistication of the analytical technique and the quality of the resulting validation, however, the measurements only deliver a given probability of being within a given concentration interval. This means that one can never be sure whether some results fall outside the acceptance level: only a majority of them will fall within these limits. From a regulatory viewpoint, this is not a fact that is clearly understood by all routine users of methods and, furthermore, by legislators.

The present article illustrates how fast the quantitative use of MS hyphenated to chromatography has evolved over the past 15 years, leading to good new quantification practices, largely inspired from the work conducted by the pharmaceutical industry. There is no doubt that these practices will continue to be improved to face the increasing complexity of regulatory constraints in the F&F domain.

## Supplementary Material

Good Quantification Practices in Mass Spectrometry of Flavours and Fragrances

## References

[1] Buttery RG, Mcfadden WH, Teranishi R, Kealy MP, Mon R 1963 Constituents of hop oil. Nature 200, 435–436. (10.1038/200435a0)

[2] Schmidt HL, Weber D, Rossmann A, Werner RA 1999 The potential of intermolecular and intramolecular isotopic correlations for authenticity control. New York, NY: Kluwer Academic/Plenum.

[3] SCCNFP. 1999 Fragrance Allergy in Consumers. (1 May 2016). See http://ec.europa.eu/health/ph_risk/committees/sccp/documents/out98_en.pdf.

[4] European_Parliament. 2003 Directive 2003/15/EC of the European Parliament and of the Council of 27 February 2003 amending Council Directive 76/768/EEC on the approximation of the laws of the Member States relating to Cosmetic Products. Official J. Eur. Union 46, 26–35.

[5] (SCCP) SCOCP. 2005 Opinion on furocoumarins in cosmetic products. (1 May 2016); SCCP Opinion 0942/05. See http://ec.europa.eu/health/ph_risk/committees/04_sccp/docs/sccp_o_036.pdf.

[6] European Commission. 2008 Regulation (EC) no 1334/2008 of the European Parliament and of the Council of 16 December 2008 on flavourings and certain food ingredients with flavouring properties for use in and on foods and amending Council Regulation (EEC) No 1601/91, Regulations (EC) No 2232/96 and (EC) No 110/2008 and Directive 2000/13/EC. Official J. Eur. Commun. L 354, 34–50.

[7] European_Parliament. 2007 Registration, Evaluation, Authorisation and Restriction of Chemicals (REACH), establishing a European Chemicals Agency, amending Directive 1999/45/EC and repealing Council Regulation (EEC) No 793/93 and Commission Regulation (EC) No 1488/94 as well as Council Directive 76/769/EEC and Commission Directives 91/155/EEC, 93/67/EEC, 93/105/EC and 2000/21/EC. Official J. Eur. Union L 396, 1–849.

[8] SCCS. 2011 Opinion on fragrance allergens in cosmetic products. (1 May 2016). See http://ec.europa.eu/health/scientific_committees/consumer_safety/docs/sccs_o_073.pdf.

[9] Kaloustian J, Mikail C, El-Moselhy T, Abou L, Portugal H 2007 GC-MS analysis of allergens in plants oils meant to cosmetics. Ol, Corps Gras, Lipides 14, 110–115. (10.1051/ocl.2007.0103)

[10] Milchard MJ et al. 2012 Application of gas-liquid chromatography to the analysis of essential oils. Perfum. Flavor. 37, 48–52.

[11] Divisova R, Vitova E, Divis P, Zemanova J, Omelkova J 2014 Validation of SPME-GC-FID method for determination of fragrance allergens in selected cosmetic products. Acta Chromatogr. 27, 8 (10.1556/AChrom.27.2015.3.8)

[12] Famiglini G, Termopoli V, Palma P, Capriotti F, Cappiello A 2013 Rapid LC-MS method for the detection of common fragrances in personal care products without sample preparation. Electrophoresis 35, 1339–1345. (10.1002/elps.201300462) 24273185

[13] Villa C, Gambaro R, Mariani E, Dorato S 2007 High-performance liquid chromatographic method for the simultaneous determination of 24 fragrance allergens to study scented products. J. Pharm. Biomed. Anal. 44, 755–762. (10.1016/j.jpba.2007.03.020) 17475438

[14] Grob KJ, Grob G, Grob K 1978 Comprehensive, standardized quality test for glass capillary columns. J. Chromatogr. 156, 1–20. (10.1016/S0021-9673(00)83120-9)

[15] Briscoe CJ, Stiles MR, Hage DS 2007 System suitability in bioanalytical LC/MS/MS. J. Pharm. Biomed. Anal. 44, 484–491. (10.1016/j.jpba.2007.03.003) 17433601

[16] European_Commission. 2010 Guidance document on pesticide residue analytical methods. SANCO. (1 May 2016); SANCO/825/00 rev. 8.1. See http://ec.europa.eu/food/plant/docs/pesticides_ppp_app-proc_guide_res_post-reg-cont-monitor.pdf.

[17] Beyer T, Diehl B, Holzgrabe U 2010 Quantitative NMR spectroscopy of biologically active substances and excipients. Bioanal. Rev. 2, 1–22. (10.1007/s12566-010-0016-8)

[18] Pauli GF 2001 qNMR—a versatile concept for the validation of natural product reference compounds. Phytochem. Anal. 12, 28–42. (10.1002/1099-1565(200101/02)12:1%3C28::AID-PCA549%3E3.0.CO;2-D) 11704959

[19] Cachet T et al. 2016 IOFI recommended practice for the use of predicted relative-response factors for the rapid quantification of volatile flavouring compounds by GC-FID. Flavour Fragr. J. 31, 191–194. (10.1002/ffj.3311)

[20] Tissot E, Rochat S, Debonneville C, Chaintreau A 2012 Rapid GC-FID quantification technique without authentic samples using predicted response factors. Flavour Fragr. J. 27, 290–296. (10.1002/ffj.3098)

[21] Ouyang G, Pawliszyn J 2008 A critical review in calibration methods for solid-phase microextraction. J. Chromatogr. A 627, 184–187. (10.1016/j.aca.2008.08.015) 18809072

[22] Chen Y, Begnaud F, Chaintreau A, Pawliszyn J 2005 Quantification of perfume compounds in shampoo using SPME. Flavour Fragr. J. 21, 822–832. (10.1002/ffj.1734)

[23] Cachet T et al. 2015 IOFI guidelines for the isolation of flavouring substances by simultaneous distillation-extraction. Flavour Fragr. J. 30, 2–4. (10.1002/ffj.3226)

[24] Chaintreau A 2000 Sample preparation: headspace techniques. In Encyclopedia of analytical chemistry (ed. Meyers RA), pp. 4229–4246. Chichester, UK: Wiley.

[25] Buszewski B, Szultka M 2012 Past, present, and future of solid phase extraction: a review. Crit. Rev. Anal. Chem. 42, 198–213. (10.1080/07373937.2011.645413)

[26] Prosen H, Kocar D 2008 Different sample preparation methods combined with LC-MS/MS and LC-UV for determination of some furocoumarin compounds in products containing citruses. Flavour Fragr. J. 23, 263–271. (10.1002/ffj.1881)

[27] Lee JH, Back YM, Lee KG, Shin HS 2008 Comparison of different solid-phase extraction methods for the analysis of heterocyclic amines from pan-fried pork meat. Korean J. Food Sci. Anim. Resour. 28, 637–644. (10.5851/kosfa.2008.28.5.637)

[28] Turesky RJ, Taylor J, Schnackenberg L, Freeman JP, Holland RD 2005 Quantitation of carcinogenic heterocyclic aromatic amines and detection of novel heterocyclic aromatic amines in cooked meats and grill scrapings by HPLC/ESI-MS. J. Agric. Food Chem. 53, 3248–3258. (10.1021/jf048290g) 15826085

[29] Chaintreau A 1999 Analysis technology. In Current topics in flavours and fragrances (ed. Swift K), pp. 97–122. Dordrecht, The Netherlands: Kluwer Academic Publishers.

[30] Rivier L 2003 Criteria for the identification of compounds by liquid chromatography-mass spectrometry and liquid chromatography-multiple mass spectrometry in forensic toxicology and doping analysis. Anal. Chim. Acta 492, 69–82. (10.1016/S0003-2670(03)00889-4)

[31] European Commission. 2002 Commission decision of 12 August 2002 implementing Council Directive 96/23/EC concerning the performance of analytical methods and the interpretation of results. Official J. Eur. Commun. L 221(2002/657/EC), 8–36.

[32] Maralikova B, Weinmann W 2004 Confirmatory analysis for drugs of abuse in plasma and urine by high-performance liquid chromatography-tandem mass spectrometry with respect to criteria for compound identification. J. Chromatogr. B 811, 21–30. (10.1016/S1570-0232(04)00642-7) 15458717

[33] Porter S, Patel R, Kay I 1996 Liquid chromatography/mass spectrometry (LC/MS)—how are the criteria for regulatory methods applied in residues analysis? In *Proc. EuroResidue III* (eds N Haagsma, A Ruiter), pp. 795–799. Utrecht, The Netherlands: Rijksuniversiteit Utrecht.

[34] Stolker AAM, Linders SHMA, van Ginkel LA, Brinkman UAT 2002 Application of the revised EU criteria for the confirmation of anabolic steroids in meat using GC/MS. Anal. Bioanal. Chem. 378, 1313–1321. (10.1007/s00216-003-2456-2) 14735271

[35] Brevard H et al. 2010 Guidelines for LC-MS identifications of flavouring substances in nature, made by the Working Group on Methods of Analysis of the International Organization of the Flavor Industry (IOFI). Flavour Fragr. J. 25, 2–3. (10.1002/ffj.1959) 20024936

[36] IOFI. 2011 Guidelines for the quantitative gas chromatography of volatile flavouring substances, from the Working Group on Methods of Analysis of the International Organization of the Flavor Industry (IOFI). Flavour Fragr. J. 26, 297–299. (10.1002/ffj.2061)

[37] IOFI. 2012 IOFI recommended practice for the quantitative analysis of volatile flavouring substances using coupled gas chromatography/mass spectrometry with selected-ion monitoring (SIM). Flavour Fragr. J. 27, 224–226. (10.1002/ffj.3092)

[38] Cachet T et al. 2015 Determination of volatile ‘restricted substances’ in flavourings and their volatile raw materials by GC-MS. Flavour Fragr. J. 30, 160–164. (10.1002/ffj.3222)

[39] Barcelo-Barrachina E, Moyano E, Puignou L, Galceran MT 2004 Evaluation of different liquid chromatography-electrospray mass spectrometry systems for the analysis of heterocyclic amines. J. Chromatogr. A 1023, 67–78. (10.1016/j.chroma.2003.10.030) 14760851

[40] Kinani S, Bouchonnet S, Magne A 2006 Détection et dosage de composés allergènes dans des compositionsde parfumerie par couplage chromatographie en phase gazeuse—spectrométrie de masse. Spectra Anal. 248, 28–38.

[41] Cabooter D, Lestremau F, Lynen F, Sandra P, Desmet G 2010 Kinetic plot method as a tool to design coupled column systems producing 100 000 theoretical plates in the shortest possible time. J. Chromatogr. A 1212, 23–34. (10.1016/j.chroma.2008.09.106) 18952223

[42] Chaintreau A et al. 2011 Collaborative validation of the quantification method for suspected allergens and test of an automated data treatment. J. Chromatogr. A 1218, 7869–7877. (10.1016/j.chroma.2011.08.072) 21945622

[43] ISO. 2005 General requirements for the competence of testing and calibration laboratories. International standard. 2005 1 May 2016; 17025. See http://www.iso.org/iso/home/store/catalogue_tc/catalogue_detail.htm?csnumber=39883.

[44] ICH. 2005 Validation of analytical procedures: text and methodology. International conference on harmonisation of technical requirements of pharmaceuticals for human use. (1 May 2016). See http://www.ich.org/fileadmin/Public_Web_Site/ICH_Products/Guidelines/Quality/Q2_R1/Step4/Q2_R1__Guideline.pdf.

[45] FDA. 2005 Guidance for Industry Validation of Analytical Procedures for Type C Medicated Feeds. (1 May 2016); 135. See http://www.fda.gov/downloads/AnimalVeterinary/GuidanceComplianceEnforcement/GuidanceforIndustry/ucm052530.pdf.

[46] ISO. 1994 Accuracy (trueness and precision) of measurement methods and results. International standard. (1 May 2016); 5725. See https://www.iso.org/obp/ui/#iso:std:iso:5725:-5:ed-1:v1:en.

[47] Feinberg M, Boulanger B, Dewe W, Hubert P 2004 New advances in method validation and measurement uncertainty aimed at improving the quality of chemical data. Anal. Bioanal. Chem. 380, 502–514. (10.1007/s00216-004-2791-y) 15365679

[48] Boulanger B, Chiap P, Dewe W, Crommen J, Hubert P 2003 An analysis of the SFSTP guide on validation of chromatographic bioanalytical methods: progresses and limitations. J. Pharm. Biomed. Anal. 32, 753–765. (10.1016/S0731-7085(03)00182-1) 12899965

[49] Hubert P et al. 2006 Quantitative analytical procedures: harmonization of the approaches. Part II—statistics. STP Pharm. Prat. 16, 30–60.

[50] Gustavo GA, Ángeles HM 2007 A practical guide to analytical method validation, including measurement uncertainty and accuracy profiles. Trends Anal. Chem. 26, 227–238. (10.1016/j.trac.2007.01.009)

[51] Hubert P et al. 2007 Harmonization of strategies for the validation of quantitative analytical procedures: a SFSTP proposal—Part III. J. Pharm. Biomed. Anal. 45, 82–96. (10.1016/j.jpba.2007.06.032) 17716847

[52] Arlenda. e-Noval (cited 19 2016). See https://www.arlenda.com/e-noval.

[53] Tranchida PQ, Maimone M, Franchina FA, Bjerk TR, Zini CA, Purcaro G, Mondello L 2016 Four-stage (low-)flow modulation comprehensive gas chromatography-quadrupole mass spectrometry for the determination of recently-highlighted cosmetic allergens. J. Chromatrogr. A 1439, 144–151. (10.1016/j.chroma.2015.12.002) 26718184

[54] Celeiro M, Lamas JP, Garcia-Jares C, Llompart M 2015 Pressurized liquid extraction-gas chromatography-mass spectrometry analysis of fragrance allergens, musks, phthalates and preservatives in baby wipes. J. Chromatogr. A 1384, 9–21. (10.1016/j.chroma.2015.01.049) 25662066

[55] Wu M-W, Yeh P-C, Chen HC, Liu L-L, Ding W-H 2013 A Microwave-assisted headspace solid-phase microextraction for rapid determination of synthetic polycyclic and nitro-aromatic musks in fish samples. J. Chin. Chem. Soc. 60, 1169–1174. (10.1002/jccs.201300029)

[56] Boyer KW, Horwitz W, Albert R 1995 Interlaboratory variability in trace element analysis. Anal. Chem. 57, 454–459. (10.1021/ac50001a031) 3838422

[57] Bassereau M et al. 2007 GC/MS quantification of suspected allergens. II. Data treatment strategies and method performances. J. Agric. Food Chem. 55, 25–31. (10.1021/jf062028l) 17199309

[58] Larger PJ, Breda M, Fraier D, Hughes H, James CA 2005 Ion-suppression effects in liquid chromatography-tandem mass spectrometry due to a formulation agent, a case study in drug discovery bioanalysis. J. Pharm. Biomed. Anal. 39, 206–216. (10.1016/j.jpba.2005.03.009) 15871916

[59] Matuszewski BK, Constanzer ML, Chavez-Eng CM 2003 Strategies for the assessment of matrix effect in quantitative bioanalytical methods based on HPLC-MS/MS. Anal. Chem. 75, 3019–3030. (10.1021/ac020361s) 12964746

[60] Mastovska K, Lehotay SJ, Anastassiades M 2005 Combination of analyte protectants to overcome matrix effects in routine GC analysis of pesticide residues in food matrixes. Anal. Chem. 77, 8129–8137. (10.1021/ac0515576) 16351165

[61] Debonneville C, Chaintreau A 2014 Online cleanup of volatile compounds in complex matrices for GCMS quantification: testing with fragranced consumer products. Flavour Fragr. J. 29, 267–276. (10.1002/ffj.3198)

[62] Hernandez F, Sancho JV, Pozo OJ 2005 Critical review of the application of liquid chromatography/mass spectrometry to the determination of pesticide residues in biological samples. Anal. Bioanal. Chem. 382, 934–946. (10.1007/s00216-005-3185-5) 15915347

[63] Annesley TM 2003 Ion suppression in mass spectrometry. Clin. Chem. 49, 1041–1044. (10.1373/49.7.1041) 12816898

[64] Chaintreau A, Joulain D, Marin C, Schmidt CO, Vey M 2003 GC/MS quantitation of fragrance compounds suspected to cause skin reactions. J. Agric. Food Chem. 51, 6398–6403. (10.1021/jf030363t) 14558753

[65] Desmedt B et al. 2014 HS-GC-MS method for the analysis of fragrance allergens in complex cosmetic matrices. Talanta 131, 444–451. (10.1016/j.talanta.2014.08.006) 25281125

[66] Rey A, Corbi E, Peres C, David N 2015 Determination of suspected fragrance allergens extended list by two-dimensional gas chromatography-mass spectrometry in ready-to-inject samples. J. Chromatogr. A 1404, 95–103. (10.1016/j.chroma.2015.05.045) 26051085

[67] Frerot E, Decorzant E 2004 Quantification of total furocoumarins in citrus oils by HPLC coupled with UV, fluorescence, and mass detection. J. Agric. Food Chem. 52, 6879–6886. (10.1021/jf040164p) 15537290

[68] Corbi E, Pérès C, David N 2014 Quantification of furocoumarins in hydroalcoholic fragrances by a liquid chromatography-high resolution/accurate mass method. Flavour Fragr. J. 29, 173–183. (10.1002/ffj.3193)

