# Supplementary material for: Good quantification practices of flavours and fragrances by mass spectrometry
Source: Philos Trans A Math Phys Eng Sci. 2016 Oct 28;374(2079):20150365. doi: 10.1098/rsta.2015.0365 (PMC5031632; doi:10.1098/rsta.2015.0365)
Supplement: Good Quantification Practices in Mass Spectrometry of Flavours and Fragrances [file rsta20150365supp1.docx]

**Good Quantification Practices in Mass Spectrometry of Flavours and Fragrances**

## Analyte identity

**Table SM-1**. Contact allergens in humans according to the SCCS list ([1](#_ENREF_1)) (an asterisk indicates the 24 allergens already listed in the SCCNFP opinion ([2](#_ENREF_2)).

| **Allergen (INCI name)** | **CAS registry number** |
| --- | --- |
| Acetylcedrene | 32388-55-9 |
| Amyl cinnamal* | 122-40-7 |
| Amyl cinnamyl alcohol* | 101-85-9 |
| Amyl salicylate | 2050-08-0 |
| Trans-anethole | 4180-23-8 |
| Anise alcohol* | 105-13-5 |
| Benzaldehyde | 100-52-7 |
| Benzyl alcohol* | 100-51-6 |
| Benzyl benzoate* | 120-51-4 |
| Benzyl cinnamate* | 103-41-3 |
| Benzyl salicylate* | 118-58-1 |
| Butylphenyl methylpropional (lilial®)* | 80-54-6 |
| Camphor | 76-22-2 /464-49-3 |
| Beta-caryophyllene (ox.) | 87-44-5 |
| Carvone | 99-49-0/6485-40-1/2244-16-8 |
| Cinnamal* | 104-55-2 |
| Cinnamyl alcohol* | 104-54-1 |
| Citral* | 5392-40-5 |
| Citronellol* | 106-22-9  1117-61-9  7540-51-4 |
| Coumarin* | 91-64-5 |
| (Damascenone ) rose ketone-4 | 23696-85-7 |
| Alpha-damascone (tmchb) | 43052-87-5  23726-94-5 |
| Cis-beta-damascone | 23726-92-3 |
| Delta-damascone | 57378-68-4 |
| Dimethylbenzyl carbinyl acetate (DMBCA) | 151-05-3 |
| Eugenol* | 97-53-0 |
| Farnesol* | 4602-84-0 |
| Geraniol* | 106-24-1 |
| Hexadecanolactone | 109-29-5 |
| Hexamethylindanopyran | 1222-05-5 |
| Hexyl cinnamal* | 101-86-0 |
| Hydroxyisohexyl, 3-cyclohexene carboxaldehyde *(hicc)** | 31906-04-4/51414-25-6 |
| Hydroxycitronellal* | 107-75-5 |
| Isoeugenol* | 97-54-1 |
| Alpha-isomethyl ionone* | 127-51-5 |
| (Dl)-limonene* | 138-86-3 |
| Linalool* | 78-70-6 |
| Linalyl acetate | 115-95-7 |
| Menthol | 1490-04-6/89-78-1/2216-51-5  78-1 / 2216-51-  5 |
| 6-methyl coumarin | 92-48-8 |
| Methyl 2-octynoate* | 111-12-6 |
| Methyl salicylate | 119-36-8 |
| 3-methyl-5-(2,2,3-trimethyl-3- cyclopentenyl)pent-4-en-2-ol | 67801-20-1 |
| Alpha-pinene and beta-pinene | 80-56-8 and 127-91-3, resp. |
| Propylidene phthalide | 17369-59-4 |
| Salicylaldehyde | 90-02-8 |
| Alpha-santalol and beta-santalol | 115-71-9 and 77-42-9, resp. |
| Sclareol | 515-03-7 |
| Terpineol (mixture of isomers) | 8000-41-7 |
| Alpha-terpineol | 10482-56-1/98-55-5 |
| Terpinolene | 586-62-9 |
| Tetramethyl acetyloctahydronaphthalenes | 54464-57-2/54464-59-4/68155-66-8/68155-67-9  68155-67-9 |
| Trimethyl-benzenepropanol (majantol) | 103694-68-4 |
| Vanillin | 121-33-5 |

**Table SM-2**. Furocoumarins of concern listed in the SCCP opinion ([3](#_ENREF_3)).

| **Furocoumarin** | **CAS registry number** |
| --- | --- |
| Byakangelicin | 482-25-7 |
| Oxypeucedanin hydrate | 2643-85-8 |
| Psoralen | 66-97-7 |
| Xanthotoxin | 298-81-7 |
| Isopimpinellin | 482-27-9 |
| Bergapten | 484-20-8 |
| Heraclenin | 35740-18-2 |
| Byakangelicol | 61046-59-1 |
| Oxypeucedanin | 737-52-0 |
| Imperatorin | 482-44-0 |
| Phellopterin | 2543-94-4 |
| Isoimperatorin | 482-45-1 |
| Epoxybergamottin | 206978-14-5 |
| 8-Geranyloxypsoralen | 71612-25-4 |
| Bergamottin | 482-46-2 |

**Table SM-3**. Volatile restricted substances in flavourings ([4](#_ENREF_4)).

| **Restricted substance** | **CAS registry number** |
| --- | --- |
| β-Asarone | 5273-86-9 |
| Camphor | 76-22-2 |
| Coumarin | 91-64-5 |
| Isoeugenol | 97-54-1 |
| Menthofuran | 17957-94-7 |
| Methylchavicol (1-allyl-4-methoxybenzene, estragole) | 140-67-0 |
| Methyleugenol (4-allyl-1,2-dimethoxy-benzene) | 93-15-2 |
| Pulegone | 89-82-7 |
| Safrole (1-allyl-3,4-methylenedioxybenzene)  Thujones (mixture of α and β ) | 94-59-7  1125-12-8d |
| α-Thujone | 546-80-5 |

## Analyte identification

### Using identification points (IPs)

**Table SM-4**. Number of IPs earned for various acquisition techniques (adapted from ([5](#_ENREF_5))).

| **Acquisition technique** | **Number of monitored ions** | **Number of IPs** |
| --- | --- | --- |
| GC-MS (EI or CI) | *n* | *n* |
| GC-MS (EI and CI) | 2 (EI) + 2 (CI) | 4 |
| LC-MS | *n* | *n* |
| GC-MS/MS | 1 precursor and 2 product ions | 4 |
| LC-MS/MS | 1 precursor and 2 product ions | 4 |
| GC-MS/MS | 2 precursor ions, each with 1 product ion | 5 |
| LC-MS/MS | 2 precursor ions, each with 1 product ion daughter | 5 |
| LC-MS/MS/MS | 1 precursor, 1 primary and 2 secondary product ions ionsionsdaughter ions | 5,5 |
| HRMS | *n* | 2 *n* |
| GC-MS and LC-MS | 2 + 2 | 4 |
| GC-MS and HRMS | 2 + 1 | 4 |

### Using the Q-value

With GC-MS techniques, an analyte is considered to be positively identified if its Q-value is at least equal to 0.90 (Equation SM-1). Below this limit, it is only tentatively identified, and the ion ratios used in the calculation of the Q-value are presumably altered by the co-elution of a compound exhibiting isobaric ions. Such an event leads to a biased (overestimated) quantification. However, the basis of this formula is unclear and it seems to combine empirical and theoretical justifications. Setting up a more robust and documented method would be extremely useful for the analytical chemistry community.

| $Q=100-\frac{\sum_{i=1}^{i=n} \left(100*\left.\left\vert r_{i}-r_{i}^{'}\right.\right\vert\right)\left(\ln\left[100r_{i}+1\right]\right)^{2}}{21.3*\sum_{i=1}^{i=n} r_{i}}$ | (Equation SM-1) |
| --- | --- |

.

| With: | $n :$ | Number of ions per compound. |  |
| --- | --- | --- | --- |
|  | *$r_{i}\text{ :}$* | Reference peak area ratio (abundance of the qualifier *i* / abundance of the quantifier) | |
|  | *$r_{i}^{'}\text{ :}$* | Observed peak area ratio. |  |

## Decisional tree

Figure SM-1 shows an example of a decisional tree that can be provided to the analyst to make a decision in the case of complex data processing. The quantitative results supported by a positive identification of the analyte (Q-value in excess of 0.90) are considered to be valid. Below this value, a co-elution is possible and the quantitative result is only tentative. Further investigations should be performed by the analyst (e.g. by using other ions without interference with a co-eluted compound).

**
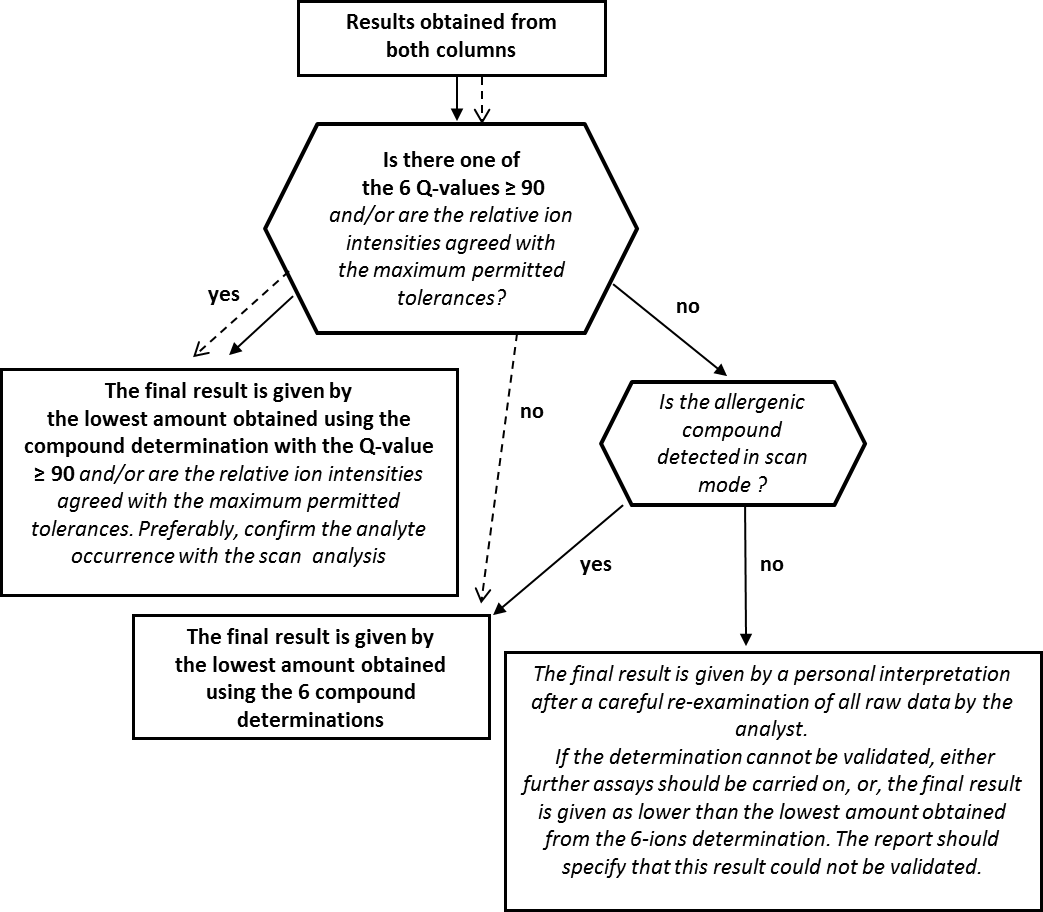
**

**Supplementary Figure SM-1:** Quantification of 24 allergens: decisional tree (whole text and continuous arrows) and automated data treatment (text in bold only and dashed arrows) used for the data treatment (Reprinted from A. Chaintreau *et al*. ([6](#_ENREF_6)), with permission of Elsevier)

## Confidence interval

To determine the confidence interval using Equation 1, *I_Conf_* = *m* ±*k*_Conf_*s*, with *m*: mean measured value at a given concentration, *k_Conf_*: coverage factor for the confidence interval and *s*: standard deviation of the measurements, the parameters are obtained according to equation SM-2:

| $k_{Conf}=\frac{t}{\sqrt{l}}$ | (Equation SM-2) |
| --- | --- |

$\mathrm{With}t$With *t*: two-tailed inverse of the student distribution for a given probability and degree of freedom, and $l$ $l$: degree of freedom (=$n$, number of replicates, if n> 30, or $n-1$ below 30).

## Accuracy profile

To determine the prediction interval under intermediate precision conditions using Equation 1, $I_{\Pr ed}=m\pm k_{\Pr ed}s_{\Pr ed}$

with *k_Pred_*: coverage factor for the prediction interval and *s_Pred_*: prediction interval standard deviation, the parameters are obtained according to Equations SM-3, SM-4, SM-5, SM-6 and SM-7 ([7](#_ENREF_7), [8](#_ENREF_8)):

| $s_{\Pr ed}=s_{R}\sqrt{1+\frac{1}{n\times J\times B^{2}}}$ | (Equation SM-3) |
| --- | --- |
| $s_{R}^{2}=s_{w}^{2}+s_{b}^{2}$ | (Equation SM-4) |

With *s_w_*^2^ and *s_b_*^2^: within-series (repeatability) and between-series variances, respectively.

| $B=\sqrt{\frac{R+1}{J\times R+1}}$ $B=\sqrt{\frac{R+1}{I\times R+1}}$ $B=\sqrt{\frac{R+1}{I\times R+1}}$ $B=\sqrt{\frac{R+1}{I\times R+1}}$ | and | $R=\frac{\sigma_{g}^{2}}{\sigma_{r}^{2}}$ $R=\frac{s_{b}^{2}}{s_{w}^{2}}$ | (Equation SM-5 and Equation SM-6) |
| --- | --- | --- | --- |

With *n* = number of series and *J* = number of replicates.

| $k_{Pred}=t_{\upsilon,\frac{1+\beta}{2}}$ | (Equation SM-7) |
| --- | --- |

$k_{\mathrm{Pred}}=t_{\upsilon,\frac{1+\beta}{2}}$ With υ: degree of freedom and β: probability of the tolerance interval.

The number of degrees of freedom υ is given by the following equation SM-8:

| $\upsilon=\frac{{(R+1)}^{2}}{\frac{{(R+\frac{1}{J})}^{2}}{n-1}+\frac{1-\frac{1}{J}}{nJ}}$ | (Equation SM-8) |
| --- | --- |

For data not coming from a combination of different variances such as intermediate precision, but strictly following a normal law (individual measurements, averages, etc.), the formula to calculate the prediction interval is much simpler, as given in equation SM-8:

$k_{\Pr ed}=t\sqrt{1+\frac{1}{l}}$ (Equation SM-8)

## References

1. SCCS. 2011 Opinion on fragrance allergens in cosmetic products. SCCS/1459/11. See <http://ec.europa.eu/health/scientific_committees/consumer_safety/docs/sccs_o_073.pdf>.

2. SCCNFP. 1999 Opinion concerning fragrance allergy in consumers. A review of the problem. SCCNFP/0017/98 Final. See <http://ec.europa.eu/health/ph_risk/committees/sccp/documents/out98_en.pdf>.

3. SCCP. 2005 Opinion on furocoumarins in cosmetic products. SCCP 0942/05. See <http://ec.europa.eu/health/ph_risk/committees/04_sccp/docs/sccp_o_036.pdf>.

4. European Commission. 2008 Regulation EC No 1334/2008 of the European Parliament and of the Council of 16 December 2008 on flavourings and certain food ingredients with flavouring properties for use in and on foods and amending Council Regulation (EEC) No 1601/91, Regulations (EC) No 2232/96 and (EC) No 110/2008 and Directive 2000/13/EC. *Official Journal of the European Communities*. L 354: 34-50.

5. European Commission. 2002 Commission decision of 12 August 2002 implementing Council Directive 96/23/EC concerning the performance of analytical methods and the interpretation of results. *Official Journal of the European Communities*. **L221** (2002/657/EC), 8-36.

6. Chaintreau A, Cicchetti E, David N, Earls A, Gimeno P, Grimaud B, Joulain D, Kupfermann N, Kruopka G, Saltron F, *et al*. 2011 Collaborative validation of the quantification method for suspected allergens and test of an automated data treatment. *J. Chromatogr. A*. **1218**, 7869-7877.

7. Feinberg M. 2010 Mise en oeuvre du profil d’exactitude. Les cahiers techniques de l'INRA. See https://[www.google.ch/url?sa=t&rct=j&q=&esrc=s&frm=1&source=web&cd=1&ved=0ahUKEwihk42QtrPMAhWHBcAKHfilDacQFggcMAA&url=https%3A%2F%2Fwww6.inra.fr%2Fcahier_des_techniques%2Fcontent%2Fdownload%2F3289%2F31792%2Fversion%2F1%2Ffile%2F27_Feinberg_Valid.pdf&usg=AFQjCNFnQiSKVypHCfKarGkEGeQu8vXtMQ](http://www.google.ch/url?sa=t&rct=j&q=&esrc=s&frm=1&source=web&cd=1&ved=0ahUKEwihk42QtrPMAhWHBcAKHfilDacQFggcMAA&url=https%3A%2F%2Fwww6.inra.fr%2Fcahier_des_techniques%2Fcontent%2Fdownload%2F3289%2F31792%2Fversion%2F1%2Ffile%2F27_Feinberg_Valid.pdf&usg=AFQjCNFnQiSKVypHCfKarGkEGeQu8vXtMQ).

8. Mee RW. 1984 b-Expectation and b-content tolerance limits for balanced one-way ANOVA random model. *Technometrics*. **26**, 251-254.
